# The Differences in Risk Perception between Practitioners in the Non-Coal-Mining Industry: Miners, Managers and Experts

**DOI:** 10.3390/toxics10100623

**Published:** 2022-10-19

**Authors:** Yuting Song, Shu Zhang

**Affiliations:** 1 School of Public Administration, Central South University, Changsha 410017, China; 2 School of Resources and Safety Engineering, Central South University, Changsha 410083, China

**Keywords:** risk perception, non-coal mine, influencing factor, risk management, human factor analysis

## Abstract

Non-coal-mining accidents occur frequently in China, and individual unsafe behaviors are the direct cause. The cognitive diversity of practitioners in the non-coal-mining industry leads to various behaviors in work and hinders communication between groups. The aim of this study is to analyze the differences in risk perception (accidents and occupational diseases) between non-coal-mining practitioners (experts, miners, and managers) and to explore the contributing factors. The questionnaire survey method was used to collect the data on risk perception and influencing factors from 402 respondents working in non-coal mines and universities in China. Project analysis and exploratory factor analysis were used for preprocessing. A t-test and linear regression analysis were used to test the significance of the differences and assess the function of the factors, respectively. Regarding risk perception, two risks both have significant differences between the three groups. With the perceptions of accidents and occupational diseases ranked from high to low, the order of the practitioners is as follows: managers (3.88), experts (3.71), miners (3.55) and experts (4.14), miners (3.90), and managers (3.88). Regarding the influencing factors, risk attitude, risk communication, educational level, enterprise trust, and occupational satisfaction have great effects on the three groups. More precisely, three groups have different important predictors. Risk attitude has the greatest impact on miners (0.290) and experts (0.369), but sensibility preference has the greatest impact on managers (0.518). In summary, cognitive discrepancies are common among non-coal-mining practitioners, but the degree of deviation varies with the type and dimension of the risk. There are six factors that have a significant impact on all practitioners, but the effect is limited by specific risks and groups.

## 1. Introduction

Modern society is faced with a variety of complex risks, not only including global risks such as climate change, environmental pollution, an energy crisis, and food safety, but also including industrial risks such as construction accidents, transportation accidents, chemical corrosion, and occupational hazards [1,2]. People are surrounded by various kinds of risks and perceive them every day. The non-coal mining industry has always been a high-danger industry. In 2021, 356 accidents and 503 deaths occurred in mines in China, representing a year-on-year decrease of 68 and 73, respectively, i.e., 16% and 12.7%. Among them, 265 accidents occurred in non-coal mines, with 325 deaths, a year-on-year decrease of 36 and 23, or 12% and 6.6%, respectively [3]. Mining accidents also occur frequently in some other countries. For example, 1444 miners died in Turkey in 2010. Although Poland’s mining technology is generally advanced, there were 311 serious accidents from 2000 to 2009 [4]. Clearly, the safety situation of non-coal mines is still severe.

The annual report of accident statistics in China shows that more than 70% of production safety accidents are human-initiated accidents [5], and there is a rising trend [6]. The accident analysis results of the DuPont company in the United States also show that 96% of all kinds of accidents that occurred in the company in the past ten years were caused by employees’ unsafe behaviors [7]. Evidently, determining a strategy to control human behavior and reduce the occurrence of human-initiated accidents is the frontier direction in the field of safety management, and it will also become the key direction of enterprise production and department supervision.

To reduce human-induced accidents, it is necessary to understand what factors determine people’s actions. Human behavior is primarily driven by perceptions rather than facts [8]. Therefore, different people may have different perceptions of the same risk, which leads to different behaviors. A relevant study in the field of nuclear power has demonstrated that experts and the public have very different cognitions of radiological hazards [9]. In the non-coal-mining industry, there are many practitioners, for example, experts, miners, and managers. Different cognitions among them may lead to discussion barriers within the industry, and different behaviors caused by different cognitions will increase the difficulty of safety management. Therefore, it is necessary to understand the perceptional differences of different groups in the non-coal-mining industry in order to promote a risk consensus.

The purpose of this study is to discuss the differences in risk perception between non-coal-mining practitioners and to analyze the factors that may cause these differences. Therefore, the risks of accidents and occupational diseases are focused on, and the research objects are technical experts, miners, and managers in the non-coal-mining industry. The main hypothesis is that different participants have different perceptions of the same risk. These findings will promote communication in the non-coal-mining industry and play a referential role for safety management.

## 2. Literature Review

### 2.1. Risk Perception of Mines

With the increasing emphasis on environmental protection worldwide, the research on mine-related environmental risk perception has also been promoted. Catalan et al. have studied the cognition of residents living around mining areas regarding manganese hazards for many years. They found that the factors influencing the cognition of Mn hazards are the community of residence, age, and having reported a chronic illness [10]. Another study found that women from two communities near the plant in the Molango mining district believe that manganese has serious effects on health and the local environment, but women of different ages have significant differences in their judgments of the degree of harm [11]. Sandra et al. developed a good scale to measure the cognition in a community chronically exposed to mining waste for a long time. The results of the scale’s analysis show that the trust of “media and authorities” is the main factor affecting people’s judgment of environmental hazards [12]. Zheng et al. constructed a psychological typhoon eye effect of mine-related environmental risk. They found that the degree of cognition of the villagers living around the mining site correlated inversely with their degree of involvement in mining activities [13].

Relevant research on miners’ cognition of occupational risk has also made progress. One explanation for frequent mining accidents is that workers struggle with fully identifying and accurately assessing hazards at the workplace. The results of an exploratory study show that job-related experience is very important for hazard identification and cognition, which determines the frequency of miners’ safe behaviors [14]. “Dreaded” and “Unknown” are the two main components of miners’ perception. The results also showed that occupational diseases are considered to be a more terrible risk type than accidents [15]. Inadequate awareness of the danger of occupational heat exposure and adaptation strategies among mine staff has led to the ineffective management of occupational heat stress. Supervisors and miners have different cognitions of the danger of heat exposure. Supervisors’ cognitions are sufficient, while miners’ cognitions are affected by their work experience and education level [16]. A cross-sectional survey highlighted the lack of knowledge of the Stampeders on the health and environmental impacts of artisanal gold mining. In addition, there are differences in cognition among workers of different working ages and positions [17]. An interesting cross-sectional questionnaire study of Swedish underground mineworkers showed that familiarity with rescue procedures, cognition of emergencies with injuries, and experience with using equipment influence the preparedness of mineworkers for a peer first response [18]. There are obvious cognitive barriers to using new safety and health technologies between workers and managers, and the differences are revealed in terms of readiness, cognition, and initial trust with respect to using various technologies [19].

To sum up, the existing studies have found that there are differences in risk perception between miners and managers and extracted some factors that affect miners’ perception. However, few studies have considered the specific differences in cognition from different types and dimensions of miners’ occupational risks. At the same time, it is also necessary to summarize the factors that may affect miners’ perception and consider the impact of these factors on the risk perception of different groups in the mining industry. On the basis of summarizing previous research results, our team extracted relevant factors that affect miners’ risk perception, namely, the organizational safety atmosphere, organizational trust, knowledge level, and risk communication. Through factor analysis, we have verified that the four effects have a positive impact on miners’ risk perception [20]. In order to discover further influencing factors, it is necessary to refer to other studies on perceptive differences and influencing factors.

### 2.2. The Differences in Risk Perception

The general public and experts have different ways of perceiving risks, and the perceptive differences between them may lead to a discussion barrier, which in turn affects the implementation of safety decisions [21]. Most relevant studies concluded that experts have significantly lower risk cognition than lay people [22]. However, some scholars indicated inconsistent results; in some cases, experts may have higher risk cognition than the public [9]. For example, experts believe that the risk of nuclear radiation leakage is higher than that of employees at nuclear power plants [23]. This result enlightened us to the fact that people might have different views on different risks in the same industry. In recent years, many scholars have carried out research on risk perception in some specific industries and achieved many valuable results. In the field of nuclear power, Perko concluded that experts and the public have different cognitions of nuclear radiation [24]; for different types of radiation hazards, the perceptive results are different [23]. In the field of construction, some scholars have elaborated the risk cognitions of different stakeholders, wherein the results have shown that when ranking risk cognitions from low to high, the order of the stakeholders is as follows: architects, contractors/safety professionals, and engineers [25]. In the field of aviation engineering, professionals and trainees have significant differences in risk perception and perform different behaviors in different flight scenarios [26]. In the chemical industry, the perception of the public is not necessarily related to the method for risk assessment used by experts, and it is a subjective and perceptual process [27]. Traffic accidents have threatened people’s safety in recent years. Studies have indicated that male drivers engage in dangerous driving behaviors more frequently than female drivers, while teen drivers engage in dangerous driving behaviors more frequently than adult drivers [28]. Over the last few years, some scholars have also examined the differences in the perceptions of public risks (non-specific industries) between different groups of people. Being overweight and obesity are strongly associated with many kinds of cancer [8]. However, there is no difference in the risk cognition of cancer between the overweight respondents and the healthy weight respondents, which is not conducive to improving public health awareness through weight management [29]. Studies on adolescents suggested that most adolescents underestimate the risks in life and there are significant differences in the cognitions of all the risks in the studies between people of different genders and ages [30]. The results of medical students’ cognition of the hazards of mobile phone use showed that female students and lower social classes have higher cognitions [31]. It is generally known that risk perception is affected by its types [23] and related interests [25]. Thus, this paper investigated two kinds of risks in non-coal mines based on different related interests: the risk of accidents and the risk of occupational diseases. Based on relevant research results of other industries, we proposed the following hypotheses regarding the non-coal-mining industry.

**H1.**  *The risk perceptions of accidents between the three groups of practitioners are significantly different, and with risk perceptions ranked from low to high, the order of the practitioners is as follows: experts, managers, and miners.*

**H2.**  *The risk perceptions of occupational diseases between the three groups of practitioners are significantly different, and with risk perceptions ranked from low to high, the order of the practitioners is as follows: experts, miners, and managers.*

### 2.3. The Influencing Factors of Risk Perception

Research related to health and cancer has demonstrated that gender and age are closely related to risk perception. In addition, age and gender have the role of regulating risk perception and adventurous behavior [28]. Therefore, personal characteristic variables (“gender”, “age”, and “educational level”) were added. As Higginbotham found that people’s cognitions of climate damage vary with time and geography [32], and the perceptive differences are also related to local policies and life experiences, we added the two factors of “working experience” and “enterprise trust”. A previous study concluded that the public is willing to accept “voluntary” dangers roughly 1000 times greater than “in-voluntary” dangers [33]. In addition, professionals and trainees have different attitudes toward different risk scenarios [26]; thus, the “risk attitude” was taken into consideration. In addition, the variable of “professional knowledge level” was added, because the state of professional knowledge will affect the cognition of relevant risks [23]. The results of an occupational survey on the hotel industry concluded that job satisfaction can regulate the relationship between work and family [34]; so, “occupational satisfaction” was taken into account. Moreover, communication is a primary way to solve dangers, so the variable of “risk communication” was also added. Finally, the public and experts describe risks in different ways [24]. In addition, some scholars in the medical field have proposed “The general population theory” and “the Expert Theory” and believe that experts and the public have different judging structures regarding risk [35,36,37]. Therefore, we considered setting three factors including “data preference”, “sensibility preference”, and “special case preference” to study the differences in risk judgment preferences of different practitioners. Perception may also vary according to different cultural backgrounds and is greatly affected by ethnicity. Research on the construction sectors in Italy found that workers from different ethnic backgrounds have differences in four dimensions of occupational hazards: behavior control, working conditions, safety atmosphere, and personal attitude [35]. Further research results showed that this different cognition regarding occupational hazards is due to cultural differences among workers of different races [36]. For example, the positive attitude towards safe actions of Eastern Europeans and Balkans indicates a kind of fatalistic acceptance of the dangerous situations at work. In addition, in the cultural conception of workers who show low risk judgment, it is considered brave to not wear protective tools. Referring to relevant research results on the influencing factors of risk perception and combining them with the characteristics of the non-coal-mining industry, we pay attention to the following influencing factors: “gender”, “age”, “educational level”, “work experience”, “enterprise trust”, “risk attitude”, “professional knowledge level”, “occupational satisfaction”, “risk communication”, “data preference”, “sensitivity preference”, and “special case preference”.

As for the hypothesis model of influencing factors, this study adopts a multiple linear regression model. The purpose of linear regression is to predict the trend, find the law, or find a suitable expression to express a trend according to the existing data. At present, most of the research on the influencing factors of risk cognition adopts the method of multiple linear regression or logistic regression analysis [31,37]. In this paper, the research on the influencing factors is divided into two parts: (1) the research on the whole group of all practitioners in the non-coal-mining industry, and (2) the research on important predictors of different practitioner groups. The reason why we study all the practitioners as a whole is because, on the one hand, this study is an exploratory study, aiming to find as many factors that can affect risk perception as possible. The multiple linear regression model can show the relationship between independent variables and dependent variables more intuitively, and find more relevant factors, which is conducive to the further study of these factors in the future. Taking all groups as samples can lead to the discovery of more influencing factors to the greatest extent. On the other hand, the reason why miners, managers, and experts can be analyzed as a group is that these three groups are stakeholders of non-coal mines and will have a certain degree of perception of occupational health and accidents in non-coal mines. Moreover, the selection of influencing factors (independent variables) in this study was implemented to select the relevant factors that may affect the risk perception of practitioners in the non-coal-mining industry through a literature review and expert consultation. The relevant literature also includes the research on the influencing factors of risk perception of stakeholders in other fields (including ordinary employees, experts, and other stakeholders). In other words, the selection of factors is not divided into different groups, so all groups should be analyzed as a whole, that is, “practitioners in the non-coal-mining industry”. The reason why we analyze the important predictors of different groups after the overall analysis is that this study is also an empirical study. We hope to contribute to the exploration of the influencing factors of risk perception of non-coal mine practitioners in theory, and hope that the results of the study can be helpful to enterprise safety management. Therefore, it is necessary to analyze the important predictors of different groups because this can provide a reference for enterprises to improve/reduce the risk perception of a specific group.

**Figure 1:**
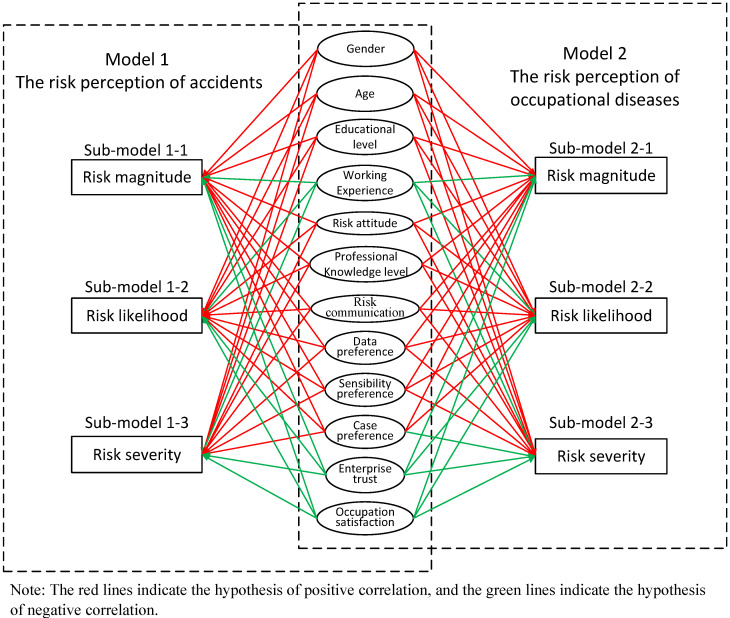
The hypothetical influencing factor model.

According to the different types of risk, two hypothetical models were proposed: the model for risk perceptions of accidents and the model for risk perceptions of occupational diseases. According to the different dimensions of each risk, three sub-models were conducted for each hypothetical model: the perceptive model of risk magnitude, risk likelihood, and risk severity. Each sub-model included the following influencing factors: gender, age, educational level, work experience, professional knowledge level, risk judgment preference (data preference, sensibility preference, and special case preference), enterprise trust, and occupational satisfaction (see Figure 1). According to the previous research results in other fields, we assume that the impact of gender is positive, that is, women’s cognition of mining risk is higher than men; age is positively correlated with risk cognition, that is, the greater the age, the higher the cognition of stakeholders [28]; in addition, the research results in relevant fields show that experts have higher cognition than employees [24]. Therefore, we assume that educational level is positively correlated with risk perception; considering that work experience is prone to overconfidence and carelessness, we assume that work experience is negatively correlated with risk perception; according to common sense, we assume that risk attitude is positively correlated with risk perception. The level of professional knowledge affects people’s understanding of danger [23]; so, we assume that the level of professional knowledge is positively correlated with risk perception; since communication will deepen people’s understanding of risk, we assume that risk communication is positively correlated with risk perception. “Data preference”, “Sensibility preference”, and “Case preference” reflect the perceptual perception trend of participants in judging danger; so, we assume that these three variables are positively correlated with risk perception. People with a high sense of enterprise trust tend to have a greater belief in the safety measures of the enterprise at work; so, we assume that there is a negative correlation between enterprise trust and risk perception. Occupational satisfaction will affect employees’ overall view of the enterprise [34]; so, we assume that occupational satisfaction is negatively correlated with risk perception.

## 3. Method

### 3.1. Participants

Non-coal mines in this study refer to underground mines that mine metal ores, radioactive ores, and other non-metallic minerals (except coal) as petrochemical raw materials, building materials, auxiliary raw materials, and refractory materials. The reasons for collecting data in lead–zinc mines are as follows: (1) Since the lead–zinc ore body is deeply buried and has many associated components, its mining methods are various. Therefore, lead–zinc mines can cover the mining risks of underground mines to the maximum extent. (2) The lead–zinc mines in China are mostly large and long-term mines, which are conducive to obtaining a large number of samples and stable data. Relevant practitioners in the non-coal-mining industry included in the study were divided into three groups: (1) miners, (2) managers of non-coal enterprises, and (3) technical experts in the non-coal-mining industry. The miners and managers of Fankou Lead Zinc Mine in Guangdong province and Panlong Lead Zinc Mine in Guangxi province were recruited to fill out both of the pre-questionnaires and the formal questionnaire. The data of experts were collected via e-mail. Based on previous studies, the scope of participants was expanded.

The group of miners included front-line workers with different positions in the two enterprises, such as blasters, electricians, ventilation workers, and locomotive drivers. The group of managers included senior managers and managers of all departments in the two enterprises. The available samples of miners and managers consisted of 220 miners and 86 managers. The group of technical experts in the non-coal-mining industry included scholars of related majors in colleges and professional technicians in the field. The usable samples of experts consisted of 96 experts from 20 universities such as Central South University in China.

### 3.2. Measures

In order to compare the risk perceptions of different groups of respondents, all participants of the three groups were asked to answer the digital questionnaire. What needs special explanation is that the questionnaire of miners and managers has two more influencing factors (independent variables) than the questionnaire of experts: enterprise trust and occupational satisfaction. Since technical experts are not affiliated with any enterprise, it is impossible to measure the enterprise trust and occupational satisfaction of experts. Independent variables in this study are factors influencing risk perception, including “gender”, “age”, “educational level”, “working experience”, “enterprise trust”, “risk attitude”, “professional knowledge level”, “occupation satisfaction”, “risk communication”, “data preference”, “sensibility preference”, and “special case preference”. Dependent variables are “risk magnitude”, “risk likelihood”, and “risk severity”, which reflect different perceptions of relevant practitioners in the non-coal-mining industry.

**Personal characteristic variables.** Personal characteristic variables included gender, age, educational level, work experience, and job position. There were two options for the gender variable: male and female. The participants were subdivided into five age categories: less than 25, 25–35, 36–45, 46–55, and more than 55. The educational level was reflected by participants’ educational background: junior high school or below, high school (technical secondary school), junior college, undergraduate, and master’s degree or above. The work experience was measured by working years: less than 1, 1–5, 6–10, 11–15, and more than 15. There were three options for the variable of job position: miner, manager, and technical expert.

**Enterprise trust.** Enterprise trust was measured by the seven-item scale of Robinson [38]. In addition, we referred to the questionnaire of Perko [23] and the questionnaire of Nie [39] and combined our items with the characteristics of non-coal-mining industry. For example, the item of “I believe my company is honest” in Robinson’s scale was replaced by the item of “I think my company actively abides by the laws and regulations of our country”. “My company is frank and honest to me” was replaced by “My company truthfully informs miners of the risks of mining accidents and occupational diseases”. The possible answers ranged from 1 (strongly disagree) to 5 (strongly agree). The total score of the scale reflected the level of enterprise trust.

**Risk attitude.** The scale of risk attitude was based on Franken’s questionnaire [40]. The questionnaire divided risk attitude into two parts: physical adventures and psychological adventures. We also referred to Zhou’s questionnaire and Hu’s questionnaire and combined the danger of non-coal-mining with our scale [41,42]. For example, “Although there are risks everywhere in life, whether or not an accident will happen depends on luck” reflected the concept of psychological adventure. “I think it is not very dangerous to stay in a place for a while where it is forbidden to stay” reflected the concept of physical adventure. Response options ranged from 1 (strongly disagree) to 5 (strongly agree). The higher the total score of the scale, the more cautious the participant’s attitude toward risks.

**Professional knowledge level.** The professional knowledge level was measured by 10 true–false questions. These questions were selected from the Chinese Certified Safety Engineer Examination, and all have official standard answers. The questions covered the knowledge of accidents and occupational diseases in the non-coal-mining industry, rules of safe operation, and methods of emergency rescue. Each correct answer counted for one point, and each wrong answer did not count for a point. The higher the total score, the higher the professional knowledge level of the participant.

**Occupational satisfaction and risk communication.** Occupational satisfaction and risk communication were directly measured with one item each. Occupational satisfaction was measured by the item “How satisfied are you with your present job”, with a scale ranging from 1 (very dissatisfied) to 5 (very satisfied). Risk communication was reflected by the item “How often do you communicate with colleagues or friends about risks in the non-coal mining industry”, with a scale ranging from 1 (never) to 5 (always).

**Data preference, sensibility preference and special case preference.** The three judging structural factors were measured with one item per factor. The answers of each item ranged from 1 (strongly disagree) to 5 (strongly agree). The three items were: “The statistics such as accident rate and death rate published by relevant departments have little significance for risk judgment”, “If an accident causes huge economic losses but no casualties, the consequences are not too serious”, and “If someone I know has been involved in an accident, I will be even more worried about a similar accident”. These three items reflect whether the participants have the habit of judging dangers through data, whether they prefer to use sensibility rather than rationality to judge the consequences of accidents, and whether they are easily affected by special cases, such as accident cases.

**Risk perception.** There were three items for each of the two risks, which were used to reflect perceptions of the magnitude, likelihood, and severity of the risks. Among them, the magnitude refers to the comprehensive assessment of risk from the likelihood and severity; the likelihood refers to the probability of occurrence of dangerous events; the severity refers to the severity of consequences caused by dangerous events. For example, “What do you think is the likelihood of production accidents in non-coal mines?” reflected the perception of the likelihood of accidents, and the answers ranged from 1 (almost unlikely) to 5 (very likely). “How do you think occupational diseases affect the lives of miners” represented the perception of the severity of occupational diseases, and the answers ranged from 1 (slight) to 5 (total incapacity to work). Moreover, “Consider both likelihood and severity, how do you think of the magnitude of accident risks in non-coal mining?” was included.

### 3.3. Data Analysis

**Pre-questionnaire.** Prior to the formal investigation, a pre-investigation was conducted to ensure the applicability and feasibility of the study. Research showed that the number of pre-survey samples should be 3–5 times the number of formal scale items [43]. This study was conducted with the aim that the final formal questionnaire would retain about 30 items; thus, the number of pre-test samples should be about 90–150. Therefore, we distributed 180 questionnaires, and finally, a total of 149 valid pre-questionnaires were collected. All data in this research were analyzed by the statistical program IBM SPSS Statistics 24. The p-value was set at 0.05 (95% confidence intervals) in all cases. Firstly, the rationality of the items was tested; then, the reliability and validity of each scale was tested through a pre-questionnaire.

The item analysis first used the critical ration method to delete the item whose t-test results were not significant and whose critical ratio was less than 3. Then, we analyzed the correlation between each item and the total score of the scale and deleted the items with the correlation coefficient less than 0.4. Finally, the method of homogeneity test was used to delete the items with the factor load less than 0.45.

The factor analysis was used to analyze the construct validity of the scale. Firstly, the KMO value was used to judge whether the scale was suitable for factor analysis. A scale with KMO value less than 0.8 is not suitable for factor analysis. Then, we checked the MSA value of each item, and the items with MSA values less than 0.5 could not be analyzed. Finally, a principal component analysis was used to delete the items with commonality less than 0.2.

The reliability of the scale was also tested after the item analysis and the validity analysis. In order to ensure the reliability of the questionnaire, the Cronbach’s Alpha value of each scale is above 0.8. Finally, it is necessary to specify that the knowledge level scale is not a Likert-scale; so, the procedure of item analysis only needed a t-test.

**Formal questionnaire.** A total of 402 valid questionnaires were collected through digital questionnaires and emails. The differences in “enterprise trust” and “occupation satisfaction” between miners and managers were analyzed via t-test. A one-way ANOVA was performed to see whether the differences in individual characteristics, influencing factors, and risk perceptions between the three groups were significant. Games–Howell post hoc test and HSD test were used for a posteriori comparisons to determine which two groups achieved significant differences. A paired sample t-test checked for significant differences within each group.

As the basis of regression analysis, the Pearson correlation test was conducted to analyze the correlation between independent variables in order to ensure that there was no strong correlation. Then, a linear regression was applied for the regression analysis of independent variables and dependent variables. According to the absolute value of regression coefficient, the influencing degree of each independent variable on dependent variables can be predicted, so as to determine whether the risk perception of practitioners can be explained by their age, educational level, work experience, risk attitude, and other factors.

## 4. Results

### 4.1. Pre-Survey

After the item analysis, the scale of enterprise trust retained all the five items. In the scale of risk attitude, the first and fifth items were deleted (see Table 1). The results of the validity analysis showed that the scale of enterprise trust only extracted one factor, and the explanations of the total variation were 72.334%, which indicated these five items could be used to explain the degree of enterprise trust. The scale of risk attitude only extracted one factor, and the explanations of the total variation were 59.151%, which indicated these six items could be used to explain the risk attitude (see Table 2).

The results of the reliability test showed that the Cronbach’s Alpha values of the two scales were both greater than 0.8, indicating that both scales had high reliability (see Table 2). In the scale concerning the professional knowledge level, the first, eighth, and tenth items were deleted. This was tested by the expert validity because the scale was in the form of true–false questions.

After the item analysis, the validity analysis, and the reliability analysis, all the inappropriate items were removed. The formal questionnaire had sufficient reliability and validity, and it could be used for the formal research. The revised questionnaire is detailed in Appendix A.

### 4.2. Differences in Characteristics

Table 3 and Table 4 present the mean values and standard deviations for the characteristics of the three different groups of respondents.

The chi-square test shows that there is no statistically significant difference in gender between the three groups of respondents (see Table 3). There is a significant difference in age between the groups (F = 4.430, *p* < 0.05), and further analysis indicates that miners (M = 2.47 and SD = 0.790) are much younger than managers (M = 2.80, SD = 0.980, and *p =* 0.015). The education level of the three groups is significantly different (F = 362.189 and *p* < 0.001), and the Games–Howell post-test reveals that the differences between any two groups are particularly significant (*p* < 0.001). The differences in work experience between the groups are significant (F = 14.259 and *p* < 0.001). In general, managers (M = 3.56 and SD = 1.252) have much more experience than miners (M = 2.98, SD = 1.047, and *p* < 0.001) and experts (M = 2.58, SD = 1.547, and *p* < 0.001). Most managers have been working in the mining industry for more than 10 years, while miners have less work experience. The risk attitude differs significantly between the three groups (F = 5.733 and *p =* 0.004), and the post hoc test implies that the miners (M = 26.291 and SD = 5.195) are more cautious about risks (p = 0.003) than the experts (M = 24.135 and SD = 4.694). However, there is no significant difference between the group of managers and the other two groups.

It can be seen from Table 4, regarding risk communication, there are significant differences between the three groups (F = 16.471 and *p* < 0.001). Miners (M = 4.18 and SD = 0.976, *p* < 0.001) and managers (M = 4.35, SD = 0.955, and *p* < 0.001) participate in risk communication more frequently than experts (M = 3.58 and SD = 1.043) in daily life. For risk judgment preferences, there is a significant difference regarding the data preference between miners (M = 4.24; SD = 1.138) and experts (M = 3.93; SD = 1.163; *p* < 0.05). The results reflect that miners prefer to use data to judge danger. For enterprise trust, there is a significant difference (t = −2.203 and *p* < 0.05) between miners (M = 22.000; SD = 4.426) and managers (M = 22.942; SD = 2.838). In general, miners have less trust in their company than managers. After a detailed analysis, we find that the distrust in the company shown by the miners is mainly due to “incomplete disclosure of information”, that is, miners think that their company’s investigation and handling of internal safety accidents is unfair (t = −2.018 *; *p* < 0.05). Furthermore, they are even less convinced that their company truthfully informs them about the danger involved in work (t = −2.893 **, *p* < 0.01). There is no significant difference with respect to occupational satisfaction, professional knowledge level, sensibility preference, or special case preference between the groups.

### 4.3. Differences in Risk Perceptions

Table 5 shows the results of a descriptive analysis for all risk perceptions. The results indicate that the participants, regardless of accidents or occupational diseases, tend to have a higher level of risk perception. The distribution of all the risk perception results shows negative skewness, indicating that most of the results concerning risk perception are on the right side of the mean. Except for the perception of accident magnitude, the excess kurtosis values of all the risk perception types are greater than 0. The results show that the distribution of the participants’ perceptions of accident magnitude is more divergent, while the distribution of their perceptions of other risks is more concentrated.

#### 4.3.1. Differences in the Risk Perception of Accidents between Groups

From Table 6, the results of the one-way ANOVA show that there are significant differences regarding the risk perception of accidents between the three groups. Since the variances of the three groups were not homogenous, a Games–Howell post hoc test was performed. Further analysis shows that managers (M_A_ = 3.88; SD_A_ = 1.162) have a higher (*p <* 0.05) perception than miners (M_A_ = 3.55; SD_A_ = 1.228), which indicates that managers pay more attention to the danger of accidents than miners. However, there is no significant difference between experts (M_A_ = 3.71; SD_A_ = 0.983) and either of the other two groups of respondents. As for the perception of the likelihood and severity of accidents, the three groups of respondents have similar perceptions without significant differences.

#### 4.3.2. Differences in the Risk Perception of Occupational Diseases between Groups

A one-way ANOVA was also performed with respect to the risk perception of occupational diseases. The differences between the three groups are significant, including the differences in risk magnitude and risk likelihood. Since the variances of the perception of risk magnitude did not pass the homogeneity test of variance, the Games–Howell test was used for the post hoc test. The variances of the perception of risk likelihood among the three groups were homogeneous; so, the HSD method was used for a post hoc test. As shown in Table 6, experts (M_O_ = 4.14; SD_O_ = 0.690) have a higher risk perception of occupational diseases than miners (M_O_ = 3.90; SD_O_ = 1.044; *p <* 0.05) and managers (M_O_ = 3.88; SD_O_ = 0.926; *p <* 0.05). However, there is no significant difference between miners and managers. The probability of occupational diseases perceived by the experts (M_O_ = 4.25; SD_O_ = 0.711) is higher than that of the managers (M_O_ = 3.99; SD_O_ = 0.901; *p <* 0.05). However, there is no significant difference in perception regarding the severity of occupational diseases between the three groups of respondents.

#### 4.3.3. Differences between the Risk Perceptions of Accidents and Occupational Diseases

Regarding the miners: there is a significant perceptive difference between the risk of accidents and the risk of occupational diseases (t = −4.738 ***, *p <* 0.001; M_A_ = 3.55; SD_A_ = 1.228; M_O_ = 3.90; SD_O_ = 1.044). There is also a significant perceptive difference between the probability of accidents and the probability of occupational diseases (t = −3.989 ***, *p <* 0.001; M_A_ = 3.89; SD_A_ = 1.131; M_O_ = 4.14; SD_O_ = 0.931).

Regarding the experts: the perception also differs significantly between the risk of accidents and the risk of occupational diseases (t = −4.694 ***, *p <* 0.001; M_A_ = 3.71; SD_A_ = 0.983; M_O_ = 4.14; SD_O_ = 0.690). There is also a significant perceptive difference between the likelihood of accidents in non-coal mines and the likelihood of workers suffering from occupational diseases (t = −3.668 ***, *p <* 0.001; M_A_ = 3.92; SD_A_ = 0.842; M_O_ = 4.25; SD_O_ = 0.711).

Regarding the managers: there is no significant difference in risk perception within the group.

### 4.4. Influencing Factors of Risk Perceptions

#### 4.4.1. Multiple Collinear Inspection

In order to understand the reason for the above differences, the influencing factors regarding the perception of the two risks were analyzed based on linear regressions. Before the formal regression analysis, the correlations between the independent variables were analyzed by Pearson correlation test, because if the correlation between independent variables is too high, it will lead to the problem of regression collinearity, resulting in inaccurate regression analysis results. Therefore, the independent variables with correlations greater than 0.7 should be deleted. In addition, the VIF value of the independent variables should be tested. If the VIF value of an independent variable is greater than five, it means that the correlation between the independent variables is too high, and it is not suitable for regression analysis. Table 7 describes the VIF values of all the independent variables and the Poisson correlation coefficients between them. The results show that the maximum correlation coefficient between the independent variables is 0.588, and the VIF values of all the independent variables are less than 5. So, there is no high correlation between the independent variables and the linear regression can be carried out (correlation coefficient > 0.7 or VIF > 5).

#### 4.4.2. Regression Analysis on Factors Influencing the Risk Perception of Accidents

With respect to Model 1, risk attitude has the greatest impact on the risk perception of accidents (see Table 8). The respondents who are cautious about dangers perceive the risk of accidents much more highly than those who are indifferent (β_M_ = 0.209 **). The respondents who often discuss occupational diseases and accidents with others, whether miners or managers, have significantly lower risk perceptions of accidents than those who seldom participate in risk communication (β_M_ = −0.172 **). In addition, the level of education also has a certain impact on the risk perception of accidents. The higher the level of education, the higher the risk perception of accidents (β_M_ = 0.130 *). Regarding likelihood and severity, risk communication and risk attitude have the greatest impact. The respondents who often participate in risk communication think the likelihood of accidents is lower than those who seldom communicate with others (β_L_ = −0.149 *). In addition, the respondents who are more cautious about dangers think the consequences of accidents in the non-coal-mining industry are more serious than those who are indifferent (β_S_ = 0.281 ***).

#### 4.4.3. Regression Analysis on Factors Influencing the Risk Perception of Occupational Diseases

Regarding Model 2, risk attitude, enterprise trust, and occupational satisfaction all have significant impacts on the risk perception of occupational diseases (see Table 8). The respondents with cautious risk attitudes perceive the danger of occupational diseases to be much higher than those with indifferent risk attitudes (β_M_ = 0.235 **). The respondents with higher trust in their enterprises have rather lower risk perceptions of occupational diseases (β_M_ = −0.175 **). The respondents with higher occupational satisfaction have lower risk perceptions of occupational diseases (β_M_ = −0.142 *). With respect to likelihood, the respondents with cautious attitudes think the likelihood of workers suffering from occupational diseases is higher than the respondents with indifferent attitudes (β_L_ = 0.183 *). In addition, the more satisfied the respondents are with their career status, the less likely they are to think that the workers will suffer from occupational diseases (β_L_ = −0.144 *). Regarding severity, the respondents who are more cautious about dangers think the effects of occupational diseases on miners are more serious (β_S_ = 0.329 ***). In addition, the respondents who are more easily influenced by special cases perceive the consequences of occupational diseases to be less serious (β_S_ = −0.117 *).

**Figure 2:**
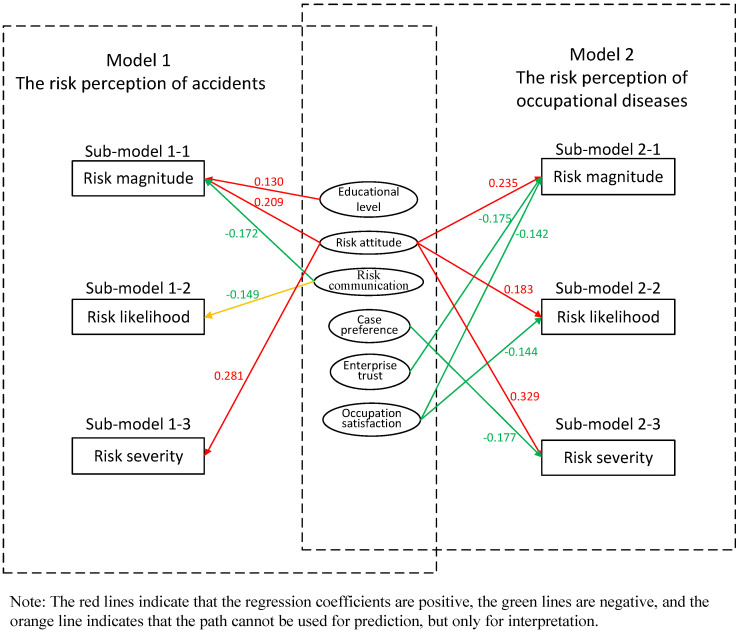
The modified influencing factor model.

To summarize, risk perceptions are influenced by a variety of factors and the extent of their impact depend on their types and dimensions. Except for the accident risk likelihood model, the R^2^ values of the other five models are all greater than 0.1 (see Table 8), and the R^2^ value of the accident risk likelihood model is 0.095, close to 0.1. If the R^2^ value is greater than 0.1, the fitting degree of the model is ideal (M L Wu, 2003). The results show that in general, the explanatory values of the six sub-models are moderately strong. The modified influencing factor model is shown in Figure 2. It should be noted that for the accident risk possibility model, although the R^2^ value is very close to 0.1 (R^2^ = 0.095), we finally chose to deny the predictability of sub-model 1-2 for the sake of the preciseness of the results. However, because risk communication has a significant correlation with the possibility of risk, we believe that sub-model 1-2 can be used to explain the perception of risk possibility (but not for prediction). In the follow-up research, we will strive to explore more influencing factors related to mining accident risk perception, so as to build an ideal model of interpretation.

#### 4.4.4. Regression Analysis on the Important Predictors of Different Groups

The predictive abilities of the influencing factors are also different between the three groups of respondents (see Table 9). For miners, the attitude toward risk is the most strongly related to the risk perception of accidents (β_AM_ = 0.213 **). Miners who are cautious about dangers perceive the risk of accidents to be much lower than the miners who are indifferent. Miners’ trust in their enterprise significantly influences their risk perception of occupational diseases as well (β_OM_ = −0.253 ***). Miners who trust their enterprise have a lower risk perception of occupational diseases than those who do not believe in their enterprise. For managers, data preference is the most important predictor (β_AM_ = −0.275 **, β_OM_ = −0.319 **). Managers who prefer to use data to judge dangers tend to underestimate dangers, whether they are accidents or occupational diseases. As for experts, risk attitude has a strong impact on the perception of accidents (β_AM_ = 0.369 ***, β_AL_ = 0.287 **). However, we failed to find any effective predictors for the experts’ risk perception of occupational diseases.

## 5. Discussion

The current study examined the differences in the perceptions of two risks in non-coal mines between three groups (the miners and the managers in non-coal-mining enterprises and the technical experts in the non-coal-mining industry). There is a significant difference in the risk perception of accidents between the three groups. However, partly different from the first hypothesis, managers have the highest risk perception and miners have the lowest risk perception. Regarding the second hypothesis, the final analysis results have proved its accuracy. This study also focused on finding factors that have a marked impact on risk perceptions and we finally determined the important influencing factors through regression analysis. Next, we will discuss the results in detail.

### 5.1. Differences in Risk Perceptions

Previous studies have shown that there are significant differences in risk cognitions between experts and the public in many industries, such as nuclear power plants [44], GM technology [45], biotechnology [46], nanotechnology [47], and chemical engineering [27]. In the non-coal-mining industry, perceptive differences between groups also exist. On the whole, there are significant perceptive differences in the risk magnitudes of both accidents and occupational diseases between the three groups. Depending on the type of risk, the results are different.

Regarding the risk perception of accidents, we hypothesized that the risk perception of miners was higher than that of managers and experts, but this has not been proved. The empirical results reveal that managers’ risk perception of accidents is much higher than that of miners, and even that of experts is higher than miners. This result is consistent with the previous results on mine risks [16]; that is, miners’ awareness of the danger of heat exposure is significantly lower than that of managers. This may be due to the fact that managers and experts pay more attention to accidents in the industry and receive more information from mass media than miners. The low risk cognition of miners may lead to unsafe behaviors in the job. This result reflects the fact that non-coal-mining enterprises have not informed miners of risks in detail, and to some extent, it also reflects the poor effects of safety training. However, the results are somewhat different from the previous research results of Slovic [22]. We believe that the difference in results may be due to the different professional requirements of employees in the nuclear power industry and mining industry. Employees and managers in the nuclear power industry both need to have a high level of nuclear power-related professional cultural literacy. As for the perceptions of the probability and severity of accidents in non-coal mines, the results are slightly different. There is no difference between the three groups, although the three groups have different cognitions regarding the risk magnitude. This result is beyond our expectations because we usually use likelihood and severity to judge dangers. We suspect that this may be due to the different ways in which different people judge danger, and this also reflects the possibility that people judge danger through more dimensions [48]. For example, previous studies have shown that “Dreaded” and “Unknown” are also important dimensions for miners to judge risks [15].

As for the risk perception of occupational diseases, whether concerning the magnitude or likelihood, the cognition of experts is much higher than that of miners and managers. We suspect that this largely depends on the fact that most miners do not fully understand the relevant knowledge of occupational diseases. A lack of knowledge makes it difficult for workers to identify the risks of occupational diseases and accurately assess them [14]. However, miners have a higher risk perception of the severity of occupational diseases than managers and experts. This can be explained by the influencing factor of the professional knowledge level. The statistical results of the professional knowledge level indicate that most miners do not know how harmful substances in minerals enter the human body, but almost all miners know that pneumoconiosis is incurable. Therefore, miners have high risk perceptions of the severity of occupational diseases but underestimate their likelihood.

The results within each group suggest that miners, managers, and experts all believe that the risk of accidents is lower than that of occupational diseases. This is similar to the research results of Ricci et al. on miners’ cognition of accidents and occupational diseases [15]. The perceptive differences in likelihood give us an explanation: all the three groups believe that the probability of accidents is lower than the likelihood of miners suffering from occupational diseases. This also shows that people prefer to attach more importance to likelihood than severity when judging risks. The intra-group analysis of the risk of occupational diseases reveals that all the three groups tend to think that miners are highly susceptible to occupational diseases, but that the impact of occupational diseases on miners is acceptable. The three groups generally believe that occupational diseases do not affect miners’ work but may lead to medical dependence. This result also proves the conclusion that occupational disease is considered to be a more terrible risk type than an accident to some extent [15].

### 5.2. Influencing Factors of Risk Perceptions

The study explored the influencing factors of risk perception of non-coal-mining industry practitioners (402 effective samples including miners, managers, and experts) and ensured the sample size of the analysis, in order to find as many factors related to risk cognition as possible, so as to facilitate the further discussion of the important predictors of risk cognition of different groups, and to have more influencing factors for other interested researchers to continue to study in the future. The results show that gender and age are not main predictors, which are different from previous studies on health risks [30]. In addition, the key findings of health risk cognitions following the COVID-19 pandemic revealed that the main factors can be broadly classified into cognitive, affective, individual, and contextual components [49]. Godovykh’s research results, like Kim’s, showed that health risk cognition is affected by individual factors such as age and gender. Regardless of industry differences, the research results on the cognition of the harm of manganese of residents around the mining area also show that gender and age are important influencing factors [10,11]. The reason for the different results may be that the main risk-bearing groups of the non-coal-mining and health industries are different. On the one hand, the majority of practitioners in the non-coal-mining industry are male, so there is no significant difference in gender. On the other hand, there is a certain correlation between age and work experience, so the regression coefficient of the age variable is low. Risk attitude is the most important predictor and has high regression coefficients in all the sub-models. This result indicates that practitioners who are more cautious in life, regardless of whether they are miners or managers, will have a higher risk cognition of accidents and occupational diseases. This is similar to the findings on COVID-19-related risk cognition. Ahmad’s research indicated that the attitude towards epidemic prevention affects the cognition of the severity of the pandemic [50]. This is also consistent with our previous research results [20]. From the perspective of safety education, miners’ risk attitudes are difficult to change. Therefore, enterprises must cultivate miners’ safety awareness by developing a good safety culture. Risk communication is strongly related to the risk cognition of accidents. Effective communication can promote miners’ improved understanding of risks, and can also effectively prevent miners from being too scared when facing dangers. In addition, enterprise trust is also a major predictor of risk perception. Miners who do not trust their enterprises may have excessively high cognitions of occupational diseases. This result is complementary to the research results of Zhang and others [51]; that is, when employees trust the enterprise more, they are more likely to show more positive thoughts and behaviors. This phenomenon can also be explained in this way: the perception of risk magnitude is not only determined by its impact; it is also influenced by a firm’s ability to treat the different risks that arise. In addition, the ability of enterprises to deal with risks is also at the core of enterprise trust, which is also an important reason behind the observed cognitive differences between the managers and miners, as they have different initial levels of trust in their respective enterprises [19]. This result reminds enterprises to pay attention to improving their image of in the minds of miners. However, this study did not find a significant relationship between knowledge level and risk perception. Furthermore, Seo’s research showed that the mastery of magnetic field knowledge affects Koreans’ cognition of the harm of mobile phones [52]. It is also inconsistent with the results of the research on mine-related heat exposure risk cognition; that is, miners’ cognition is affected by their work experience and knowledge level [16]. We speculate that this may be due to the fact that miners’ judgment of danger depends more on experience than professional knowledge.

In addition, this study further explored the important predictors of risk perception of different groups in order to provide reference for non-coal-mining enterprises or non-coal mine regulatory authorities to improve the risk cognition of specific groups (miners, managers, or experts) in the future. The results show that the regression coefficients of the different groups are also quite different. The same influencing factor may have different effects on different groups, which implies that if we want to improve the risk awareness of specific groups, we need to consider different influencing factors to make our actions more efficient.

### 5.3. Limitations and Future Research

In the present study, we examined different groups’ perceptions of risk magnitude, risk likelihood. and risk severity. However, in fact, risk has many dimensions, not only the likelihood and the severity; the other dimensions of risk need to be studied in the future, such as “Dreaded” and “Unknown” [15]. In terms of influencing factors, this study considered the variables of personal characteristics, enterprise trust, risk attitude, risk communication, and other factors, and explored the linear relationship between these factors and risk perception. However, these factors may interact with each other, so the relationship between them may be a more complex regression relationship. For example, we failed to explain why in this study, the level of knowledge had no significant impact on miners’ risk perception, while in the study of Nunfam [16], the differences in the knowledge level regarding the distribution of adaptation strategies of occupational heat stress were significant. Therefore, this study is only a preliminary exploration of the impact mechanism, and our research group will further study the impact mechanism of risk perception in the future. Finally, the sample of the present study was collected from China. Although the initial design of the study considered the impact of race and culture, due to the limited number of samples, this study failed to collect sample data from different races. In the field of construction, studies have shown that race and culture have a significant impact on workers’ occupational risk cognition [35,36]. However, the mechanism of their impact on miners’ risk perception has not been fully confirmed. The influence of cultural and ethnic differences on risk perception needs to be considered. So, in the future, we will conduct in-depth research on foreign non-coal-mining enterprises.

## 6. Conclusions

Accidents and occupational diseases are both risks that threaten the health and lives of miners. This study reveals that different groups of practitioners have significant differences regarding their perceptions of the two risks. Previous studies on risk cognition mostly focused on the difference between experts and the public, but this research focuses on miners, managers, and experts. In general, managers have higher a risk perception of accidents than miners and experts. However, the three groups of respondents have a similar perception of the likelihood and severity of accidents. This result demonstrates that a risk may encompass multiple dimensions, not only likelihood and the severity. Regarding occupational diseases, with risk perceptions ranked from low to high, the order of the respondents is as follows: experts, miners, and managers. Furthermore, the probability of occupational diseases perceived by experts is higher than that of managers. As for the influencing factors of risk perception, risk attitude, risk communication, educational level, enterprise trust, occupational satisfaction, and data preference are all important predictors. It is worth noting that the predictive abilities of different influencing factors change with the groups: risk attitude and enterprise trust are the strongest predictors of the risk perception of miners; for managers, sensibility preference is the most important predictor; for experts, risk attitude has a significant impact on the risk perception of accidents. However, we did not determine the predictors of the risk perception of occupational diseases for experts.

## Figures and Tables

**Table 1:** Item analysis of corporate trust and risk attitude.

Scale	Item	Extreme Group Method	Relevance between Item and Total Score		Commonality Analysis			Unqualified Indicators	Remarks
		CR	Item	Corrected Item	Correctedα	Communality	Factor Loadings		
Corporate trust	A1	100.450 ***	0.778 ***	0.660	0.899	0.595	0.771	0	Retain
	A2	110.407 ***	0.881 ***	0.793	0.872	0.761	0.872	0	Retain
	A3	110.125 ***	0.920 ***	0.856	0.857	0.841	0.917	0	Retain
	A4	90.523 ***	0.851 ***	0.777	0.878	0.744	0.863	0	Retain
	A5	110.500 ***	0.814 ***	0.721	0.888	0.676	0.822	0	Retain
Standard		≥30.000	≥40.000	≥40.000	≤0.901	≥0.200	≥0.450		
Risk attitude	B1	70.183 ***	0.369 ***	0.254	0.818	0.147	0.383	5	Delete
	B2	60.948 ***	0.712 ***	0.600	0.777	0.521	0.722	0	Retain
	B3	100.863 ***	0.698 ***	0.553	0.784	0.442	0.665	0	Retain
	B4	90.370 ***	0.707 ***	0.583	0.779	0.478	0.691	0	Retain
	B5	80.997 ***	0.581 ***	0.350	0.834	0.209	0.457	2	Delete
	B6	60.779 ***	0.821 ***	0.757	0.760	0.756	0.869	0	Retain
	B7	60.206 ***	0.759 ***	0.665	0.768	0.671	0.819	0	Retain
	B8	40.968 ***	0.727 ***	0.654	0.779	0.614	0.783	0	Retain
Standard		≥30.000	≥40.000	≥40.000	≤0.809	≥0.200	≥0.450		

CR (Critical value): This is the boundary value between acceptance domain and rejection domain. If the difference of CR of an item is not statistically significant (CR < 3), the item is considered to be unable to distinguish the reaction degree of different subjects and should be deleted. *** means the results are significant at 0.001 alpha level.

**Table 2:** Reliability and validity analysis of enterprise trust and risk attitude.

Scale	Item	KMO	Bartlett’s Test	MSA	Communality	Unqualified Indicators	Remarks	Cronbach’s Alpha
Enterprise trust	A1	0.861	0.000	0.906	0.595	0	Retain	0.901
	A2			0.850	0.761	0	Retain	
	A3			0.808	0.841	0	Retain	
	A4			0.881	0.744	0	Retain	
	A5			0.893	0.676	0	Retain	
Risk attitude	B1	0.822	0.000	0.864	0.549	0	Retain	0.848
	B2			0.814	0.458	0	Retain	
	B3			0.870	0.496	0	Retain	
	B4			0.764	0.755	0	Retain	
	B5			0.766	0.674	0	Retain	
	B6			0.915	0.618	0	Retain	
Standard		≥0.8	<0.05	≥0.5	≥0.2			≥0.8

KMO (Kaiser–Meyer–Olkin measure of sampling adequacy): The closer the KMO value is to 1, the stronger the correlation between variables, and the more suitable the original variables are for factor analysis; the closer the KMO value is to 0, the weaker the correlation between variables, and the less suitable the original variables are for factor analysis. MSA (measure of sampling adequacy): This constitutes the comparison value of all correlation coefficients and net correlation coefficients related to the measurement variable. The larger the coefficient is, the better the correlation.

**Table 3:** Characteristics of different groups of respondents.

		Gender	Age	Educational level	Working Experience	Risk Attitude	Professional Knowledge Level
Miners	Mean	1.22	2.47	2.32	2.98	26.291	4.873
	SD	0.414	0.790	0.916	1.047	5.195	1.144
Managers	Mean	1.16	2.80	3.30	3.56	25.232	5.058
	SD	0.371	0.980	0.946	1.252	6.119	1.392
Experts	Mean	1.16	2.54	4.98	2.58	24.135	5.031
	SD	0.365	0.994	0.144	1.574	4.694	1.192
Total	Mean	1.19	2.56	3.16	3.01	25.549	4.950
	SD	0.394	0.892	1.356	1.275	5.357	1.212

**Table 4:** Characteristics of different groups of respondents.

		Risk Communication	Data Preference	Sensibility Preference	Special Case Preference	Enterprise Trust	Occupational satisfaction
Miners	Mean	1.82	4.24	4.07	2.10	22.000	3.82
	SD	0.976	1.138	1.268	1.312	4.427	1.064
Managers	Mean	1.65	3.97	4.14	2.00	22.942	3.99
	SD	0.955	1.376	1.248	1.168	2.838	0.927
Experts	Mean	3.58	3.93	1.89	4.02	-	-
	SD	1.043	1.163	0.993	1.005		
Total	Mean	2.20	4.10	3.56	2.53	22.265	3.87
	SD	1.255	1.204	1.525	1.471	4.062	1.029

**Table 5:** Descriptive statistics of the risk perceptions.

	Number	Range	Minimum	Maximum	Mean	SD	Skewness		Excess Kurtosis	
							Statistics	SE	Statistics	SE
Accident magnitude	402	4	1	5	30.66	10.165	−0.857	0.122	−0.107	0.243
Accident likelihood	402	4	1	5	30.92	10.076	−0.934	0.122	0.278	0.243
Accident severity	402	4	1	5	40.06	0.673	−10.753	0.122	60.964	0.243
Occupational disease magnitude	402	4	1	5	30.95	0.949	−10.062	0.122	10.164	0.243
Occupational disease likelihood	402	4	1	5	40.13	0.879	−10.123	0.122	10.556	0.243
Occupational disease severity	402	4	1	5	30.66	0.953	−10.044	0.122	10.308	0.243

**Table 6:** Risk perceptions of different groups of respondents.

		Miners		Managers		Experts	
		Mean	SD	Mean	SD	Mean	SD
Accident	Magnitude	3.55	1.228	3.88	1.162	3.71	0.983
	Likelihood	3.89	1.131	4.00	1.168	3.92	0.842
	Severity	4.07	0.676	3.99	0.711	4.11	0.630
Occupational disease	Magnitude	3.90	1.044	3.88	0.926	4.14	0.690
	Likelihood	4.14	0.931	3.99	0.901	4.25	0.711
	Severity	3.69	1.063	3.59	0.899	3.66	0.708

**Table 7:** Correlations between independent variables and the VIF value of each variable.

		Gender	Age	Educational Level	Work experience	Risk Attitude	Professional Knowledge level	Risk Communication	Data Preference	Sensibility Preference	Special Case Preference	Enterprise Trust	Occupational satisfaction
VIF		1.235	1.483	1.126	1.728	1.964	1.237	1.384	1.705	1.502	1.045	1.316	1.308
Gender	Pc	1											
	Sig.(2−tailed)												
	N	402											
Age	Pc	−0.148 **	1										
	Sig.(2−tailed)	0.003											
	N	402	402										
Educational level	Pc	−0.012	−0.121 *	1									
	Sig.(2−tailed)	0.805	0.015										
	N	402	402	402									
Work experience	Pc	−0.346 ***	0.569 ***	−0.132 **	1								
	Sig.(2−tailed)	0.000	0.000	0.008									
	N	402	402	402	402								
Risk attitude	Pc	0.010	0.119 *	−0.197 ***	0.139 **	1							
	Sig.(2−tailed)	0.838	0.017	0.000	0.005								
	N	402	402	402	402	402							
Professional knowledge level	Pc	−0.137 **	0.238 ***	−0.024	0.341 ***	0.241 ***	1						
	Sig.(2−tailed)	0.006	0.000	0.634	0.000	0.000							
	N	402	402	402	402	402	402						
Risk communication	Pc	0.097	−0.066	0.496 ***	−0.142 **	−0.133 **	0.051	1					
	Sig.(2−tailed)	0.051	0.186	0.000	0.004	0.007	0.308						
	N	402	402	402	402	402	402	402					
Data preference	Pc	0.000	0.141 **	−0.117 *	0.102 *	0.588 ***	0.149 **	−0.057	1				
	Sig.(2−tailed)	0.996	0.005	0.019	0.042	0.000	0.003	0.254					
	N	402	402	402	402	402	402	402	402				
Sensibility preference	Pc	0.011	0.020	−0.467 ***	0.155 **	0.380 ***	0.076	−0.434 ***	0.267 ***	1			
	Sig.(2−tailed)	0.822	0.685	0.000	0.002	0.000	0.129	0.000	0.000				
	N	402	402	402	402	402	402	402	402	402			
Special case preference	Pc	−0.083	0.074	0.393 ***	−0.072	−0.043	−0.016	0.472 ***	0.033	−0.293 ***	1		
	Sig.(2−tailed)	0.099	0.136	0.000	0.150	0.389	0.752	0.000	0.508	0.000			
	N	402	402	402	402	402	402	402	402	402	402		
Enterprise trust	Pc	−0.029	−0.043	−0.101	−0.084	0.115 *	−0.009	−0.349 ***	0.207 ***	0.107	−0.056	1	
	Sig.(2−tailed)	0.615	0.456	0.078	0.145	0.044	0.872	0.000	0.000	0.062	0.327		
	N	306	306	306	306	306	306	306	306	306	306	306	
Occupational satisfaction	Pc	0.040	0.124 *	−0.103	−0.015	−0.022	0.021	−0.355 ***	0.140 *	−0.054	−0.046	0.427 ***	1
	Sig.(2−tailed)	0.481	0.030	0.071	0.798	0.706	0.716	0.000	0.014	0.344	0.425	0.000	
	N	306	306	306	306	306	306	306	306	306	306	306	306

Pc—Pearson correlation; VIF—Variance inflation factor. *** Correlation is significant at the 0.001 level (2-tailed). ** Correlation is significant at the 0.01level (2-tailed). * Correlation is significant at the 0.05 level (2-tailed).

**Table 8:** Influencing factors of risk perceptions of accidents and occupational diseases.

Influencing Factors	Model 1—The Risk Perception of Accidents						Model 2—The Risk Perception of Occupational Diseases					
	Magnitude		Likelihood		Severity		Magnitude		Likelihood		Severity	
	Beta(β)	t	Beta(β)	t	Beta(β)	t	Beta(β)	t	Beta(β)	t	Beta(β)	t
Gender	−0.003	−0.043	−0.043	−0.675	−0.022	−0.356	−0.100	−1.635	−0.006	−0.100	−0.042	−0.690
Age	0.025	0.374	0.091	1.337	0.068	1.048	0.062	0.944	0.007	0.097	0.090	1.360
Educational level	0.130 *	2.249	0.002	0.042	0.042	0.746	0.034	0.601	−0.086	−1.473	0.069	1.206
Work experience	0.046	0.646	0.067	0.918	−0.077	−1.099	−0.106	−1.497	0.087	1.203	−0.036	−0.503
Risk attitude	0.209 **	2.733	0.058	0.739	0.281 ***	3.758	0.235 **	3.103	0.183 *	2.371	0.329 ***	4.349
Professional knowledge level	0.093	1.535	−0.007	−0.108	0.037	0.617	0.113	1.886	0.063	1.024	0.092	1.531
Risk communication	−0.172 **	−2.687	−0.149*	−2.276	−0.030	−0.475	−0.092	−1.458	−0.046	−0.719	−0.080	−1.260
Data preference	−0.104	−1.454	−0.044	−0.609	0.077	1.096	−0.136	−1.923	−0.092	−1.270	−0.099	−1.393
Sensibility preference	0.083	1.231	0.116	1.685	0.077	1.175	0.073	1.099	0.051	0.755	0.029	0.435
Case preference	−0.028	−0.491	−0.057	−0.979	−0.098	−1.773	−0.045	−0.806	−0.101	−1.767	−0.117*	−2.097
Enterprise trust	−0.038	−0.596	−0.095	−1.454	0.032	0.505	−0.175 **	−2.769	−0.119	−1.845	−0.106	−1.672
Occupational satisfaction	−0.113	−1.743	−0.125	−1.882	−0.109	−1.726	−0.142 *	−2.219	−0.144 *	−2.203	−0.063	−0.988
	R^2^ = 0.13		R^2^ = 0.095		R^2^ = 0.171		R^2^ = 0.153		R^2^ = 0.119		R^2^ = 0.151	

Note: Linear regression analysis and Dependent variables: risk perception of accidents and risk perception of occupational diseases. *** *p <* 0.001; ** *p <* 0.01; * *p <* 0.05.

**Table 9:** Important predictors of different groups for the risk perceptions of accidents occupational diseases.

		Miners	Managers	Experts
Accidents	Magnitude	Risk attitude (0.213 **)	Sensibility preference (0.518 ***)	Risk attitude (0.369 ***)
		Educational level (0.136 **)	Data preference (−0.275 *)	
	Likelihood	Work experience (0.153 *)	Sensibility preference (0.458 ***)	Special case preference (0.298 **)
			Data preference (−0.296 **)	Risk attitude (0.287 **)
	Severity	Data preference (0.191 *)	Risk attitude (0.514 *)	Data preference (0.313 **)
		Risk attitude (0.183 *)		
Occupational diseases	Magnitude	Enterprise trust (−0.253 ***)	Risk attitude (0.424 ***)	-
		Risk attitude (0.216 ***)	Data preference (−0.319 **)	
	Likelihood	Work experience (0.190 **)	Risk attitude (0.293 **)	-
		Occupational satisfaction (−0.186 **)		
	Severity	Risk attitude (0.290 ***)	Risk attitude (0.502 ***)	-
		Enterprise trust (−0.158 *)	Data preference (−0.295 *)	

Note: *** *p <* 0.001; ** *p <* 0.01; * *p <* 0.05.

## Data Availability

The data that support the findings of this study are available from the corresponding author, S.Z., upon reasonable request.

## Appendix A. Formal Questionnaire

The questionnaire on the differences of risk perceptions and the influencing factors among non-coal-mining practitioners

*Dear Mr./Ms.*

*Hello! First of all, thank you for your cooperation in this survey. The purpose of this questionnaire is to study the differences of risk perceptions among non-coal-mining practitioners and analyze the related factors influencing risk perceptions. Each of your answers will have a key impact on the results; please fill in your true thoughts!*

*This questionnaire will not disclose your name and contact information, and will not be used for any commercial purposes. It is only used for academic research. It is strictly confidential and will not affect you and your enterprise. Please rest assured!*

*Thank you for your patience!*

Part I: Basic information

Note: Please select (tick the option) or fill in according to your actual situation.

1. Your gender? □ male     □ female 2. What’s your age? □ less than 25  □ 25–35  □ 36–45  □ 46–55  □ more than 55 years old 3. What’s your educational background? □ junior high school or below   □ senior high school (technical secondary school) □ junior college   □ undergraduate   □ master’s degree or above 4. How long have you worked in the mining industry? □ less than 1 year  □ 1–5 years  □ 6–10 years  □ 11–15 years  □ more than 15 years 5. What’s your position now? □ miner  □ manager  □ technical expert.

Part II: Risk perceptions

Note: Please tick the corresponding options according to your real feelings and thoughts. Please answer the questions one by one in order to avoid omission.

1. Consider both likelihood and severity, what do you think the magnitude of accident risks in non-coal mining is? □ very low  □ low  □ not clear  □  high  □ very high 2. What do you think the likelihood of production accidents in non-coal mines is? □ almost unlikely  □ not likely  □ not clear  □ likely  □ very likely 3. How serious are the consequences of mine accidents? □ almost negligible  □ slight  □ not clear  □ serious  □ destructive 4. Consider both likelihood and severity, what do you think the magnitude of occupational disease risks in non-coal mines is? □ very low  □ low  □ not clear  □  high  □ very high 5. What do you think the likelihood of miners suffering from occupational diseases is? □ almost unlikely  □ not likely  □ not clear  □ likely □ very likely 6. How do you think occupational diseases affect the lives of miners? □ slight        □ temporary       □ no influence on work but medical dependence □ loss of part of labor capacity      □ total incapacity to work

Part III: The influencing factors of risk perceptions

Note: Please tick the corresponding options according to your real feelings and thoughts. Please answer the questions one by one in order to avoid omission (“1” means strongly disagree; “2” means disagree; “3” means not clear; “4” means agree; “5” means strongly agree).

Questions	1	2	3	4	5
I think my company actively abides by the laws and regulations of our country.					
My company truthfully informs miners of the risks of mining accidents and occupational diseases.					
My company rewards safe operation and punishes risky operation fairly and reasonably.					
My company’s investigation and handling of internal safety accidents is completely open and transparent.					
My company can effectively control accidents and ensure the safety of employees.					
Although there are risks everywhere in life, whether an accident will happen or not depends on luck.					
Most of the risks in my daily life don’t hurt me too much.					
I think it is not very dangerous to stay in a place for a while where it is forbidden to stay.					
I think some small risks can be properly ignored in order to complete the work as soon as possible.					
I think some safety procedures are too cumbersome and have little effect.					
I think that as long as the operation skill is good, no special work permit is required.					
The statistics such as accident rate and death rate published by relevant departments have little significance for risk judgment.					
If an accident causes huge economic losses but no casualties, the consequences are not too serious.					
If someone I know has been involved in an accident, I will be even more worried about a similar accident.					
How satisfied are you with your present job? □ very dissatisfied □ dissatisfied □ not clear □ satisfied □ very satisfied					
How often do you communicate with colleagues or friends about risks in the non-coal-mining industry? □ never □ seldom □ occasionally □ often □ always					

Part IV: Professional knowledge

**Questions**	**T/F**
The appearance of water droplets on the wall, the decrease in air temperature, and the appearance of fog are all the omens of water penetration accidents.	
When going up and down the raise, two or more people can share a ladder.	
In the haulage shaft, you can take a tramcar to go up and down the shaft.	
For the working face with poor ventilation, the fan should be started for 5 min before entering.	
When a fire or poisoning accident occurs in non-coal mines, the self-rescuer should be worn, and when the inhaled air is dry or hot, the self-rescue device should be taken out quickly.	
Pneumoconiosis can be prevented and cured.	
Harmful substances in minerals are mainly absorbed by the human body through respiratory tract, stomach, and skin.	

*The questionnaire is over, and thank you for your cooperation!*
